# Evaluating the performance of memory type logarithmic estimators using simple random sampling

**DOI:** 10.1371/journal.pone.0278264

**Published:** 2022-12-15

**Authors:** Shashi Bhushan, Anoop Kumar, Amani Alrumayh, Hazar A. Khogeer, Ronald Onyango

**Affiliations:** 1 Department of Statistics, University of Lucknow, Lucknow, U.P., India; 2 Department of Statistics, Amity University, Lucknow, India; 3 Department of Mathematics, College of Science, Northern Border University, Arar, Saudi Arabia; 4 Department of Mathematical Sciences, College of Applied Sciences, Umm Al-Qura University, Makkah, Saudi Arabia; 5 Department of Applied Statistics, Financial Mathematics and Acturial Science, Jaramogi Oginga Odinga University of Science and Technology, Bondo, Kenya; Mustansiriyah University - College of Science, IRAQ

## Abstract

In survey research, various types of estimators have been suggested that consider only the current sample information to compute the unknown population parameters. Therefore, we utilize the past sample information along with the current sample information in the form of hybrid exponentially weighted moving averages to suggest the memory type logarithmic estimators for time-based surveys. The expression of the mean square error of the suggested estimators is determined to the first order of approximation. A relative comparison of the suggested estimators with the existing estimators is performed and efficiency conditions are obtained. Further, a simulation study is accomplished using a hypothetically rendered population and a real data illustration to improve the theoretical results. The results of the simulation study and the real data application exhibit that the consideration of past and current sample information meliorates the efficiency of the suggested estimators.

## 1 Introduction

In sample surveys, usually, the information on an auxiliary variable, namely, population mean $\bar{X}$, population standard deviation *S*_*x*_, population correlation coefficient *ρ*_*xy*_, population coefficient of skewness *β*_1_(*x*), population coefficient of kurtosis *β*_2_(*x*) etc., are known in advance which are positively or negatively correlated with the study variable. The utilization of this information helps to enhance the efficiency of the suggested estimators. [1] introduced the ratio estimator when the auxiliary information is positively correlated with the study variable as

$$\begin{array}{c} {\bar{y}}_{r}=\bar{y}(\frac{\bar{X}}{\bar{x}}) \end{array}$$ (1)

[2] investigated the classical product estimator when the auxiliary information is negatively correlated with the study variable as

$$\begin{array}{c} {\bar{y}}_{p}=\bar{y}(\frac{\bar{x}}{\bar{X}}) \end{array}$$ (2)

where $\bar{x}=n^{-1}\sum_{i=1}^{n}x_{i}$ and $\bar{y}=n^{-1}\sum_{i=1}^{n}y_{i}$ are the sample means of the auxiliary variable *x* and study variable *y* respectively.

The mean square error (*MSE*) of the above ratio and product estimators is given as

$$\begin{array}{c} MSE({\bar{y}}_{r})=f{\bar{Y}}^{2}\left(C_{y}^{2}+C_{x}^{2}-2\rho_{xy}C_{x}C_{y}\right) \end{array}$$ (3)

$$\begin{array}{c} MSE({\bar{y}}_{p})=f{\bar{Y}}^{2}\left(C_{y}^{2}+C_{x}^{2}+2\rho_{xy}C_{x}C_{y}\right) \end{array}$$ (4)

where *f* = 1/*n*, $\bar{Y}$ is the population mean of study variable *y*, *C*_*x*_ and *C*_*y*_ are the population coefficient of variations of the auxiliary and study variables, respectively.

[3] suggested a class of logarithmic type estimator as

$$\begin{array}{c} {\bar{y}}_{bk}=\bar{y}{\left\{1+\text{log}\;(\frac{\bar{x}}{\bar{X}})\right\}}^{\alpha } \end{array}$$ (5)

where *α* is a duly opted scalar.

The *MSE* of the estimator ${\bar{y}}_{bk}$ up to the first order of approximation is given by

$$\begin{array}{c} MSE({\bar{y}}_{bk})=f{\bar{Y}}^{2}\left(C_{y}^{2}+\alpha^{2}C_{x}^{2}+2\alpha \rho_{xy}C_{x}C_{y}\right) \end{array}$$ (6)

The minimum *MSE* at optimum value of *α*_(*opt*)_ = −*ρ*_*xy*_(*C*_*y*_/*C*_*x*_) is given by

$$\begin{array}{c} MSE({\bar{y}}_{bk})=f{\bar{Y}}^{2}C_{y}^{2}\left(1-\rho_{xy}^{2}\right) \end{array}$$ (7)

Over the recent years, utilizing the current sample information, a large number of ratio, product, regression and exponential type estimators consist of different auxiliary information have been suggested by several authors. [4] utilized the known correlation coefficient for estimating the population mean. [5] suggested a general estimation procedure of population mean using known values of population parameters. [6] introduced estimation of entropy with application to test of fit under RSS. [7] suggested the estimation of proportion in presence of tie using RSS. [8] discussed interval estimation of *P*(*X* < *Y*) using RSS. [9] suggested estimation of a symmetric distribution function in multistage RSS, whereas [10] examined a new goodness of fit tests for the Cauchy distribution. [11] suggested improved modified ratio estimators using robust regression estimators. [12] suggested some efficient classes of estimators under stratified random sampling, whereas [13] suggested some novel class of estimators of population mean under ranked set sampling (RSS). [14, 15] developed some log type class of population mean estimators under RSS. [16] suggested quantile regression ratio type estimators of population mean using complete and partial auxiliary information, whereas [17] proposed quantile robust regression type estimators based on minimum covariance determinant for estimating population mean. [18] suggested two auxiliary variable based robust regression ratio type estimators of the population mean. [19] analyzed the covid-19 risk using an exponential estimators. [20] developed double sampling based robust ratio estimators in the presence of outliers. [21] considered an improved class of robust ratio estimators by using the minimum covariance determinant estimation. [22] investigated robust regression type estimators to estimate population mean using simple and two-stage sampling. [23] introduced robust ratio type estimators for estimating population mean under simple random sampling. [24] examined the Poisson regression ratio estimators for population mean using double sampling. [25] proposed compromised imputation based mean estimators using robust quantile regression method.

It has been well established that the consideration of auxiliary information helps to meliorate the efficacy of the estimators. Due to this reason, only the current sample ${\bar{y}}_{t}$ at time *t* is utilized to obtain the knowledge of the study and auxiliary variables. The efficiency of the estimator can further be enhanced by utilizing the knowledge of the current and the previous samples such as ${\bar{y}}_{t-1}$ from time *t* − 1, ${\bar{y}}_{t-2}-1$ from time *t* − 2 and all this. It becomes essential in situations when a particular time interval based survey such as monthly or annual is performed. [26] introduced memory type estimators for population mean utilizing exponentially weighted moving averages (EWMA) for time scaled surveys under SRS, whereas [27] developed the memory type product and ratio estimators for population mean utilizing hybrid exponentially weighted moving averages (HEWMA) for time-based surveys under SRS. Later on, [28] suggested memory type product and ratio estimators of population mean in stratified sampling, whereas [29] investigated the memory type product and ratio estimators of population mean utilizing ranked-based sampling schemes. [30] studied a memory type shrinkage estimator of population mean in the quality control process. [31] suggested the ratio and product type estimators for estimating population mean for time-based survey. Recently, [32] suggested memory-type ratio and product estimators for population variance utilizing EWMA statistics for time-scaled surveys.

In the theory of survey sampling, the classical estimators are based on functional forms, like chains, exponential functions, and linear combinations. However, the construction of estimators using the logarithmic function is novel. Development of advance data analysis tools has enhanced the computational limitations of these functions. In the real world, logarithms are utilized for the measurement of earthquakes on Richter scales, acidity on pH scale, and sound on decibels. In addition, logarithms can also be utilized to measure exponential decay and growth, like interest rates, bacterial growth in a Petri dish, and radioactive decay in carbon dating. Some real life examples are also given in Section 6 and a numerical study is performed. Therefore, it is important to study logarithmic type estimators for the estimation of population parameters and this is the motivation behind the construction of the suggested class of estimators. This study aims to proffer a memory type logarithmic estimator based on the knowledge of the past as well as current sample information. To achieve this aim, the HEWMA statistic is considered in this study.

The plan of this article is described in few sections. In Section 2, *HEWMA* statistic is described along with the review of the existing memory type estimators along with their properties. The developed memory type logarithmic estimator is given in Section 3 along with its properties. In Section 4, a comparative study is performed by comparing the *MSE* expressions of the suggested and existing estimators. A simulation study is accomplished in Section 5 and a real data illustration is given in Section 6. The conclusion is outlined in Section 7.

## 2 Reviews of estimators based on *HEWMA* statistic

[33, 34], respectively, introduced the concept of the EWMA statistic and the HEWMA statistic for efficiently detecting the changes in the fundamental process mean in control charting. Let *X*_1_, *X*_2_, …., *X*_*n*_ be an independent and identically distributed random variables from which the sequence *HE*_1_, *HE*_2_, …, *HE*_*n*_ are defined by utilizing the undermentioned recurrence expressions.

$$\begin{array}{c} E_{t}=\lambda_{2}{\bar{X}}_{t}+\left(1-\lambda_{2}\right)E_{t-1}\;0<\lambda_{2}\leq 1 \end{array}$$ (8)

$$\begin{array}{c} HE_{t}=\left(1-\lambda_{1}\right)HE_{t-1}+\lambda_{1}E_{t}\;0<\lambda_{1}\leq 1 \end{array}$$ (9)

where λ_*i*_, *i* = 1, 2 are duly chosen scalars. Also, *E*_*t*_ and *HE*_*t*_ denote the *EWMA* and *HEWMA* statistic, respectively. The initial values of these statistics are considered as expected mean which can be estimated from a pilot survey. In this study, it is considered as zero i.e. *HE*_0_ = *Et*_0_ = 0. The mean and variance of *HE*_*t*_ statistic were computed by [34]. Later on, [35] observed that the expressions of mean and variance derived by [34] were incorrect, then [35] provided the correct expressions of mean and variance of *HE*_*t*_ statistic as

$$\begin{array}{c} E(HE_{t})=\bar{Y} \end{array}$$ (10)

$$\begin{array}{c} V(HE_{t})=\frac{\lambda_{1}^{2}\lambda_{2}^{2}}{{\left(\lambda_{1}-\lambda_{2}\right)}^{2}}\{\begin{array}{c} \frac{{\left(1-\lambda_{1}\right)}^{2}\left(1-{\left(1-\lambda_{1}\right)}^{2t}\right)}{1-{\left(1-\lambda_{1}\right)}^{2}}+\frac{{\left(1-\lambda_{2}\right)}^{2}\left(1-{\left(1-\lambda_{2}\right)}^{2t}\right)}{1-{\left(1-\lambda_{2}\right)}^{2}}\\ -\frac{2\left(1-\lambda_{1}\right)\left(1-\lambda_{2}\right)\left(1-{\left(1-\lambda_{1}\right)}^{t}{\left(1-\lambda_{2}\right)}^{t}\right)}{1-\left(1-\lambda_{1}\right)\left(1-\lambda_{2}\right)} \end{array}\}\frac{\sigma_{y}^{2}}{n} \end{array}$$ (11)

where *t* ≥ 1, $\bar{Y}$ and $\sigma_{y}^{2}$ are the mean and variance of the study variable. The limiting form of the variance is expressed by

$$\begin{array}{c} V(HE_{t})=\frac{\lambda_{1}^{2}\lambda_{2}^{2}}{{\left(\lambda_{1}-\lambda_{2}\right)}^{2}}\{\frac{{\left(1-\lambda_{1}\right)}^{2}}{1-{\left(1-\lambda_{1}\right)}^{2}}+\frac{{\left(1-\lambda_{2}\right)}^{2}}{1-{\left(1-\lambda_{2}\right)}^{2}}-\frac{2\left(1-\lambda_{1}\right)\left(1-\lambda_{2}\right)}{1-\left(1-\lambda_{1}\right)\left(1-\lambda_{2}\right)}\}\frac{\sigma^{2}}{n} \end{array}$$ (12)

$$\begin{array}{c} V(HE_{t})=\frac{\lambda_{1}^{2}\lambda_{2}^{2}}{{\left(\lambda_{1}-\lambda_{2}\right)}^{2}}\delta \frac{\sigma_{y}^{2}}{n} \end{array}$$ (13)

where $\delta =\frac{{\left(1-\lambda_{1}\right)}^{2}}{1-{\left(1-\lambda_{1}\right)}^{2}}+\frac{{\left(1-\lambda_{2}\right)}^{2}}{1-{\left(1-\lambda_{2}\right)}^{2}}-\frac{2\left(1-\lambda_{1}\right)\left(1-\lambda_{2}\right)}{1-\left(1-\lambda_{1}\right)\left(1-\lambda_{2}\right)}$.

[27] considered the *HEWMA* statistic to propose the memory type ratio and product estimators. The *HEWMA* statistic for the study variable is given by

$$\begin{array}{c} E_{ty}=\lambda_{2}{\bar{y}}_{t}+\left(1-\lambda_{2}\right)E_{ty-1} \end{array}$$ (14)

$$\begin{array}{c} Z_{t}=\lambda_{1}E_{ty}+\left(1-\lambda_{1}\right)Z_{t-1} \end{array}$$ (15)

and the *HEWMA* statistic for the auxiliary variable is given by

$$\begin{array}{c} E_{tx}=\lambda_{2}{\bar{x}}_{t}+\left(1-\lambda_{2}\right)E_{tx-1} \end{array}$$ (16)

$$\begin{array}{c} Q_{t}=\lambda_{1}E_{tx}+\left(1-\lambda_{1}\right)Q_{t-1} \end{array}$$ (17)

The statistic *Z*_*t*_ and *Q*_*t*_ are unbiased for population mean $\bar{Y}$ and $\bar{X}$ respectively.

Utilizing the statistic *Z*_*t*_ and *Q*_*t*_, [27] suggested the memory type ratio and product estimators under simple random sampling as

$$\begin{array}{c} {\bar{y}}_{r}^{M}=Z_{t}(\frac{\bar{X}}{Q_{t}}) \end{array}$$ (18)

$$\begin{array}{c} {\bar{y}}_{p}^{M}=Z_{t}(\frac{Q_{t}}{\bar{X}}) \end{array}$$ (19)

To deduce the properties of the suggested estimators, we consider the following annotations.

$Z_{t}=\bar{Y}\left(1+e_{0}\right)$ , $Q_{t}=\bar{X}\left(1+e_{1}\right)$ such that *E*(*e*_0_) = *E*(*e*_1_) = 0 and $E\left(e_{0}^{2}\right)=f\delta \left\{{\left(\lambda_{1}\lambda_{2}\right)}^{2}/{\left(\lambda_{1}-\lambda_{2}\right)}^{2}\right\}C_{y}^{2}$, $E\left(e_{1}^{2}\right)=f\delta \left\{{\left(\lambda_{1}\lambda_{2}\right)}^{2}/{\left(\lambda_{1}-\lambda_{2}\right)}^{2}\right\}C_{x}^{2}$ *E*(*e*_0_*e*_1_) = *fδ*{(λ_1_λ_2_)^2^/(λ_1_ − λ_2_)^2^}*ρ*_*xy*_*C*_*x*_*C*_*y*_.

The *MSE* of the ratio and product estimators to the first order of approximation are given by

$$\begin{array}{c} MSE({\bar{y}}_{r}^{M})=f{\bar{Y}}^{2}\frac{{\left(\lambda_{1}\lambda_{2}\right)}^{2}}{{\left(\lambda_{1}-\lambda_{2}\right)}^{2}}\delta \left(C_{x}^{2}+C_{y}^{2}-2\rho_{xy}C_{x}C_{y}\right) \end{array}$$ (20)

$$\begin{array}{c} MSE({\bar{y}}_{p}^{M})=f{\bar{Y}}^{2}\frac{{\left(\lambda_{1}\lambda_{2}\right)}^{2}}{{\left(\lambda_{1}-\lambda_{2}\right)}^{2}}\delta \left(C_{x}^{2}+C_{y}^{2}+2\rho_{xy}C_{x}C_{y}\right) \end{array}$$ (21)

It is to be noted that the value of *δ* will be replaced by *δ*_1_ which is given by

$$\begin{array}{c} \delta_{1}=\{\frac{{\left(1-\lambda_{1}\right)}^{2}\left(1-{\left(1-\lambda_{1}\right)}^{2t}\right)}{1-{\left(1-\lambda_{1}\right)}^{2}}+\frac{{\left(1-\lambda_{2}\right)}^{2}\left(1-{\left(1-\lambda_{2}\right)}^{2t}\right)}{1-{\left(1-\lambda_{2}\right)}^{2}}-\frac{2\left(1-\lambda_{1}\right)\left(1-\lambda_{2}\right)\left(1-{\left(1-\lambda_{1}\right)}^{t}{\left(1-\lambda_{2}\right)}^{t}\right)}{1-\left(1-\lambda_{1}\right)\left(1-\lambda_{2}\right)}\} \end{array}$$ (22)

## 3 Suggested memory type logarithmic estimators

The logarithmic function has a few important characteristics and furnishes an essential role in various dimensions of scientific and non-scientific disciplines. In this article, we have developed the logarithmic relationship between the study variable *Y* and the auxiliary variable *X* for computing the population mean $\bar{Y}$. The developed estimators would perform in situations where the variables *Y* and *X* are logarithmically associated. Motivated by the works of [26, 27], we propose the memory type logarithmic estimator using the *HEWMA* statistic as

$$\begin{array}{c} {\bar{y}}_{bk}^{M}=Z_{t}{\left[1+\text{log}(\frac{Q_{t}}{\bar{X}})\right]}^{\beta } \end{array}$$ (23)

where *β* is a duly opted scalar.

Using the notations described in the preceding section, we rewrite the estimator ${\bar{y}}_{bk}^{M}$ in terms of *e*′*s* as

$$\begin{array}{c} {\bar{y}}_{bk}^{M}-\bar{Y}=\bar{Y}(e_{0}+\beta e_{1}-\beta e_{1}^{2}+\frac{\beta^{2}}{2}e_{1}^{2}+\beta e_{0}e_{1}) \end{array}$$ (24)

Squaring and taking expectation both sides of (24), we get the *MSE* of the estimator ${\bar{y}}_{bk}^{M}$ to the first order of approximation as

$$\begin{array}{c} MSE\left({\bar{y}}_{bk}^{M}\right)=f{\bar{Y}}^{2}\frac{\lambda_{1}^{2}\lambda_{2}^{2}}{{\left(\lambda_{1}-\lambda_{2}\right)}^{2}}\delta \left(C_{y}^{2}+\beta^{2}C_{x}^{2}-2\beta \rho_{xy}C_{x}C_{y}\right) \end{array}$$ (25)

Minimizing the $MSE({\bar{y}}_{bk}^{M})$ with respect to *β*, we get

$$\begin{array}{c} \beta_{\left(opt\right)}=-\rho_{xy}\frac{C_{y}}{C_{x}} \end{array}$$ (26)

The minimum *MSE* at the optimum value of *β* is given by

$$\begin{array}{c} MSE\left({\bar{y}}_{bk}^{M}\right)=f{\bar{Y}}^{2}\frac{\lambda_{1}^{2}\lambda_{2}^{2}}{{\left(\lambda_{1}-\lambda_{2}\right)}^{2}}\delta C_{y}^{2}\left(1-\rho_{xy}^{2}\right) \end{array}$$ (27)

The above *MSE* expression is important to perform the comparative study in the next section.

## 4 Comparative study

To derive the efficiency conditions, we perform a comparative study by comparing the minimum *MSE* of the developed memory type logarithmic estimator with the minimum *MSE* of the existing conventional and memory type estimators.

1. By comparing the minimum *MSE* of the developed memory type logarithmic estimator from (27) with the minimum *MSE* of the usual mean estimator, we get
  $$\begin{array}{c} \rho_{xy}^{2}>1-\frac{{\left(\lambda_{1}-\lambda_{2}\right)}^{2}}{\delta \lambda_{1}^{2}\lambda_{2}^{2}} \end{array}$$ (28)
2. By comparing the minimum *MSE* of the developed memory type logarithmic estimator from (27) with the minimum *MSE* of the classical ratio estimator from (3), we get
  $$\begin{array}{c} \rho_{xy}^{2}>1-\frac{{\left(\lambda_{1}-\lambda_{2}\right)}^{2}}{\delta C_{y}^{2}\lambda_{1}^{2}\lambda_{2}^{2}}\left(C_{y}^{2}+C_{x}^{2}-2\rho_{xy}C_{x}C_{y}\right) \end{array}$$ (29)
3. By comparing the minimum *MSE* of the developed memory type logarithmic estimator from (27) with the minimum *MSE* of the classical product estimator from (4), we get
  $$\begin{array}{c} \rho_{xy}^{2}>1-\frac{{\left(\lambda_{1}-\lambda_{2}\right)}^{2}}{\delta C_{y}^{2}\lambda_{1}^{2}\lambda_{2}^{2}}\left(C_{y}^{2}+C_{x}^{2}+2\rho_{xy}C_{x}C_{y}\right) \end{array}$$ (30)
4. By comparing the minimum *MSE* of the developed memory type logarithmic estimator from (27) with the minimum *MSE* of the conventional logarithmic type estimator from (7), we get
  $$\begin{array}{c} \frac{\delta \lambda_{1}^{2}\lambda_{2}^{2}}{{\left(\lambda_{1}-\lambda_{2}\right)}^{2}}<1 \end{array}$$ (31)
5. By comparing the minimum *MSE* of the developed memory type logarithmic estimator from (27) with the minimum *MSE* of the memory type ratio estimator from (20), we get
  $$\begin{array}{c} \rho_{xy}^{2}>1-\frac{\left(C_{y}^{2}+C_{x}^{2}-2\rho_{xy}C_{x}C_{y}\right)}{C_{y}^{2}} \end{array}$$ (32)
6. By comparing the minimum *MSE* of the developed memory type logarithmic estimator from (27) with the minimum *MSE* of the memory type product estimator from (21), we get
  $$\begin{array}{c} \rho_{xy}^{2}>1-\frac{\left(C_{y}^{2}+C_{x}^{2}+2\rho_{xy}C_{x}C_{y}\right)}{C_{y}^{2}} \end{array}$$ (33)

Under the above efficiency conditions, the suggested memory type logarithmic estimator dominates the existing conventional and memory type estimators. Furthermore, these conditions are substantiated with a simulated study and a real data illustration.

## 5 Simulation study

To illustrate the effectiveness of the suggested memory type logarithmic estimators against the existing conventional and memory type ratio and product estimators, we conduct a simulation study over an artificially rendered bivariate normal population of size *N* = 1000 units using R software with parameters $\bar{X}=40$, $\bar{Y}=30$, *σ*_*x*_ = 6 and *σ*_*y*_ = 5. Using 40,000 iterations, the *MSE* and relative efficiency (*RE*) of various estimators (*t*) are computed by utilizing the undermentioned expressions:

$$\begin{array}{c} MSE(t)=\frac{1}{40000}\sum_{i=1}^{40000}{\left(t-\bar{Y}\right)}^{2} \end{array}$$ (34)

$$\begin{array}{c} RE=\frac{MSE(\bar{y})}{MSE(t)} \end{array}$$ (35)

The performance of the developed memory type logarithmic estimator is studied by considering various amounts of correlation coefficients *ρ*_*xy*_ = ±0.1, ±0.3, ±0.5, ±0.7, ±0.9 and sample sizes *n* = 10, 20, 50, 100, 200, 300. The performance of the existing and suggested memory type estimators is also examined by considering various amounts of λ_2_ = 0.05, 0.25, 0.5, 0.75, 1 and a fixed value of λ_1_ = 0.1. The simulation results are revealed from Tables 1 to 4 for different values of *ρ*_*xy*_, *n* and λ_2_. From the simulation results, we draw the interpretation in point-wise fashion as follows:

1. From Tables 1 and 2, it has been observed that the suggested memory type logarithmic estimator ${\bar{y}}_{bk}^{M}$ dominates the usual mean estimator $\bar{y}$, classical ratio estimator ${\bar{y}}_{r}$, logarithmic estimator ${\bar{y}}_{bk}$, memory type ratio estimator ${\bar{y}}_{r}^{M}$ in terms of lesser *MSE* and greater *RE* for different amount of *ρ*_*xy*_ = +0.1, +0.3, +0.5, +0.7, +0.9.
2. From Tables 3 and 4, it has been observed that the suggested memory type logarithmic estimator ${\bar{y}}_{bk}^{M}$ dominates the usual mean estimator $\bar{y}$, classical product estimator ${\bar{y}}_{p}$, logarithmic estimator ${\bar{y}}_{bk}$, memory type product estimator ${\bar{y}}_{p}^{M}$ in terms of lesser *MSE* and greater *RE* for different amount of *ρ*_*xy*_ = −0.1, −0.3, −0.5, −0.7, −0.9.
3. From Table 1, it has been observed that the *MSE* of the suggested memory type logarithmic estimator ${\bar{y}}_{bk}^{M}$ decreases as the values of λ_2_ increase, whereas a reverse inclination can be seen from the results of Table 2 reported in terms of *RE*.
4. The similar inclination can be seen from the results of Tables 3 and 4 reported, respectively, in terms of *MSE* and *RE*.
5. From Tables 1 and 3, it is noticed that the *MSE* of the various estimators decreases as the sample size increase for each value of *ρ*_*xy*_, whereas from Tables 2 and 4, it is seen that the *RE* increases as the sample size increases for each value of *ρ*_*xy*_.
6. From Tables 1 and 3, it is also observed that the *MSE* of various estimators decreases as the values of correlation coefficient increases, whereas from Tables 2 and 4, it is seen that the *RE* increases as the values of *ρ*_*xy*_ increases.

**Table 1:** *MSE* of existing and suggested estimators for various values of *ρ*_*xy*_, λ and *n*.

					λ_2_ = 0.05		λ_2_ = 0.25		λ_2_ = 0.5		λ_2_ = 0.75		λ_2_ = 1	
*ρ*_*xy*_	*n*	${\bar{y}}_{m}$	${\bar{y}}_{r}$	${\bar{y}}_{bk}$	${\bar{y}}_{r}^{M}$	${\bar{y}}_{bk}^{M}$	${\bar{y}}_{r}^{M}$	${\bar{y}}_{bk}^{M}$	${\bar{y}}_{r}^{M}$	${\bar{y}}_{bk}^{M}$	${\bar{y}}_{r}^{M}$	${\bar{y}}_{bk}^{M}$	${\bar{y}}_{r}^{M}$	${\bar{y}}_{bk}^{M}$
0.1	10	2.4735	4.1201	2.4487	0.0711	0.0422	0.1596	0.0948	0.1905	0.1132	0.2056	0.1222	0.2168	0.1288
	20	1.2389	2.0515	1.2265	0.0354	0.0211	0.0794	0.0475	0.0948	0.0567	0.1024	0.0612	0.1079	0.0645
	50	0.4958	0.8186	0.4908	0.0141	0.0084	0.0317	0.0190	0.0378	0.0227	0.0408	0.0245	0.0430	0.0258
	100	0.2479	0.4086	0.2454	0.0070	0.0042	0.0158	0.0095	0.0189	0.0113	0.0203	0.0122	0.0215	0.0129
	200	0.1238	0.2041	0.1225	0.0035	0.0021	0.0079	0.0047	0.0094	0.0056	0.0101	0.0061	0.0107	0.0064
	500	0.0495	0.0816	0.0490	0.0014	0.0008	0.0031	0.0019	0.0037	0.0022	0.0040	0.0024	0.0042	0.0025
0.3	10	2.4259	3.2596	2.2076	0.0562	0.0381	0.1263	0.0855	0.1507	0.1021	0.1627	0.1101	0.1715	0.1161
	20	1.2116	1.6042	1.1026	0.0276	0.0190	0.0621	0.0427	0.0742	0.0509	0.0800	0.0550	0.0844	0.0580
	50	0.4852	0.6371	0.4415	0.0109	0.0076	0.0246	0.0171	0.0294	0.0204	0.0318	0.0220	0.0335	0.0232
	100	0.2425	0.3174	0.2207	0.0054	0.0038	0.0123	0.0085	0.0146	0.0102	0.0158	0.0110	0.0167	0.0116
	200	0.1211	0.1584	0.1102	0.0027	0.0019	0.0061	0.0042	0.0073	0.0050	0.0080	0.0055	0.0083	0.0058
	500	0.0484	0.0633	0.0441	0.0010	0.0007	0.0025	0.0017	0.0030	0.0020	0.0031	0.0022	0.0033	0.0023
0.5	10	2.4174	2.4017	1.8130	0.0414	0.0313	0.0930	0.0702	0.1110	0.0838	0.1198	0.0904	0.1264	0.0954
	20	1.2065	1.1629	0.9049	0.0200	0.0156	0.0450	0.0350	0.0537	0.0418	0.0580	0.0451	0.0612	0.0476
	50	0.4831	0.4576	0.3623	0.0080	0.0062	0.0177	0.0140	0.0211	0.0167	0.0228	0.0180	0.0240	0.0190
	100	0.2415	0.2272	0.1811	0.0040	0.0030	0.0088	0.0070	0.0105	0.0083	0.0113	0.0090	0.0119	0.0095
	200	0.1206	0.1132	0.0904	0.0020	0.0015	0.0043	0.0035	0.0052	0.0041	0.0056	0.0045	0.0059	0.0047
	500	0.0482	0.0452	0.0362	0.0008	0.0006	0.0018	0.0014	0.0020	0.0016	0.0023	0.0018	0.0023	0.0019
0.7	10	2.4234	1.5464	1.2359	0.0266	0.0213	0.0599	0.0478	0.0715	0.0571	0.0771	0.0616	0.0813	0.0650
	20	1.2097	0.7243	0.6169	0.0125	0.0106	0.0280	0.0239	0.0335	0.0285	0.0361	0.03079	0.0381	0.0324
	50	0.4842	0.2791	0.2469	0.0048	0.0042	0.0108	0.0095	0.0129	0.0114	0.0139	0.0123	0.0146	0.0129
	100	0.2420	0.1376	0.1234	0.0024	0.0020	0.0053	0.0047	0.0063	0.0057	0.0069	0.0060	0.0072	0.0064
	200	0.1209	0.0683	0.0616	0.0012	0.0010	0.0026	0.0023	0.0031	0.0028	0.0034	0.0030	0.0036	0.0032
	500	0.0484	0.0272	0.0246	0.0005	0.0004	0.0010	0.0009	0.0012	0.0010	0.0014	0.0012	0.0014	0.0012
0.9	10	2.4451	0.6952	0.4645	0.0120	0.0080	0.0269	0.0180	0.0321	0.0214	0.0347	0.0231	0.0365	0.0244
	20	1.2218	0.2882	0.2321	0.0050	0.0040	0.0111	0.0089	0.0133	0.0107	0.0143	0.0115	0.0151	0.0122
	50	0.4887	0.1017	0.0928	0.0018	0.0016	0.0040	0.0035	0.0048	0.0042	0.0050	0.0045	0.0053	0.0048
	100	0.2444	0.0486	0.0464	0.0009	0.0006	0.0019	0.0017	0.0022	0.0020	0.0024	0.0021	0.0026	0.0024
	200	0.1221	0.0237	0.0232	0.0006	0.0002	0.0009	0.0008	0.0011	0.0009	0.0012	0.0011	0.0013	0.0010
	500	0.0488	0.0093	0.0092	0.0004	0.0001	0.0004	0.0001	0.0004	0.0001	0.0005	0.0004	0.0005	0.0004

**Table 2:** *RE* of existing and suggested estimators for various values of *ρ*_*xy*_, λ and *n*.

				λ_2_ = 0.05		λ_2_ = 0.25		λ_2_ = 0.5		λ_2_ = 0.75		λ_2_ = 1	
*ρ*_*xy*_	*n*	${\bar{y}}_{r}$	${\bar{y}}_{bk}$	${\bar{y}}_{r}^{M}$	${\bar{y}}_{bk}^{M}$	${\bar{y}}_{r}^{M}$	${\bar{y}}_{bk}^{M}$	${\bar{y}}_{r}^{M}$	${\bar{y}}_{bk}^{M}$	${\bar{y}}_{r}^{M}$	${\bar{y}}_{bk}^{M}$	${\bar{y}}_{r}^{M}$	${\bar{y}}_{bk}^{M}$
0.1	10	0.6003	1.0101	34.7890	58.6137	15.4981	26.0917	12.9842	21.8507	12.0306	20.2414	11.4091	19.2041
	20	0.6038	1.0101	34.9971	58.7156	15.6032	26.0821	13.0685	21.8500	12.0986	20.2434	11.4819	19.2077
	50	0.6056	1.0101	35.1631	59.0238	15.6403	26.0947	13.1164	21.8414	12.1519	20.2367	11.5302	19.2170
	100	0.6067	1.0101	35.4142	59.0238	15.6898	26.0947	13.1164	21.9380	12.2118	20.3196	11.5302	19.2170
	200	0.6065	1.0106	35.3714	58.9523	15.6708	26.3404	13.1702	22.1071	12.2574	20.2950	11.5700	19.3437
	500	0.6066	1.0102	35.3571	61.8750	15.9677	26.0526	13.3783	22.5000	12.3750	20.6250	11.7857	19.8000
0.3	10	0.7442	1.0988	46.1197	63.6719	19.2074	28.3730	16.0975	23.7600	14.9102	22.0336	14.1451	20.8949
	20	0.7552	1.0988	43.8985	63.7684	19.5104	28.3747	16.3288	23.8035	15.1450	22.290	14.3554	20.8896
	50	0.7615	1.0989	44.5137	63.8421	19.7235	28.3742	16.5034	23.7843	15.2578	22.0545	14.4835	20.9137
	100	0.7640	1.0987	44.9074	63.8157	19.7154	28.5294	16.6095	23.7745	15.3481	22.0454	14.5209	20.9051
	200	0.7645	1.0989	44.8518	63.7368	19.8524	28.8333	16.5890	24.2200	15.1375	22.0181	14.5903	20.8793
	500	0.0633	1.0975	48.4000	69.1428	19.3600	28.4705	16.1333	24.2000	15.6129	22.0000	14.6666	21.0434
0.5	10	1.0065	1.3333	58.3913	77.2332	25.9935	34.4358	21.7783	28.8472	20.1786	26.7411	19.1250	25.3396
	20	1.0374	1.3332	60.3250	77.3397	26.8111	34.4714	22.4674	28.8636	20.8017	26.7516	19.7140	25.3466
	50	1.0557	1.3334	60.3875	77.9193	27.2937	34.5071	2.8957	28.9281	21.1885	26.8388	20.1291	25.4263
	100	1.0629	1.3335	60.3750	80.5000	27.4431	34.5000	23.0000	29.0963	21.3716	26.8333	20.2941	25.4210
	200	1.0653	1.3340	60.3000	80.4000	28.0465	34.4571	23.1923	29.4146	21.5357	26.8000	20.4406	25.6595
	500	1.0663	1.4785	60.2500	80.3333	26.7777	34.4285	24.1000	30.1250	20.9565	26.7777	20.9565	25.3684
0.7	10	1.5671	1.9608	91.1052	113.7746	40.4574	50.6987	33.8937	42.4413	31.4319	39.3409	29.8081	37.2830
	20	1.6701	1.9609	96.7760	114.1226	43.2035	50.6150	36.1104	42.4456	33.5096	39.4039	31.7506	37.3364
	50	1.7348	1.9611	100.8750	115.2857	44.8333	50.9684	37.5348	42.4736	34.8345	39.3658	33.1643	37.5348
	100	1.7587	1.9611	100.8333	121.0000	45.6603	51.4893	38.4126	42.4561	35.0724	40.3333	33.6111	37.8125
	200	1.7701	1.9626	100.75	120.9000	46.5000	52.5652	39.0000	43.1785	35.5588	40.3000	33.5833	37.7812
	500	1.7794	1.9674	96.8000	121.000	48.4000	53.7777	40.3333	48.4000	34.5714	40.3333	34.5714	40.3333
0.9	10	3.5171	5.2639	203.7583	305.6375	90.8959	135.8388	76.1713	114.2570	70.4639	105.8484	66.9890	100.2090
	20	4.2394	5.2641	244.3600	305.4500	110.0720	137.2808	91.8646	114.1869	85.4405	106.2434	80.9139	100.1475
	50	4.8053	5.2661	271.5000	305.4375	122.1750	139.6285	101.8125	116.3571	97.7400	108.6000	92.2075	101.8125
	100	5.0288	5.2672	271.5555	407.3333	128.6315	143.7647	111.0909	122.2000	101.8333	116.3809	94.0000	101.8333
	200	5.1518	5.2629	297.8048	488.400	135.6666	152.625	111.0000	135.6666	101.7500	111.0000	93.9230	122.1000
	500	5.2473	5.3043	122.0000	488.0000	122.0000	488.0000	122.0000	488.0000	97.6000	122.0000	97.6000	122.0000

**Table 3:** *MSE* of existing and suggested estimators for various values of *ρ*_*xy*_, λ and *n*.

					λ_2_ = 0.05		λ_2_ = 0.25		λ_2_ = 0.5		λ_2_ = 0.75		λ_2_ = 1	
*ρ*_*xy*_	*n*	${\bar{y}}_{m}$	${\bar{y}}_{p}$	${\bar{y}}_{bk}$	${\bar{y}}_{p}^{M}$	${\bar{y}}_{bk}^{M}$	${\bar{y}}_{p}^{M}$	${\bar{y}}_{bk}^{M}$	${\bar{y}}_{p}^{M}$	${\bar{y}}_{bk}^{M}$	${\bar{y}}_{p}^{M}$	${\bar{y}}_{bk}^{M}$	${\bar{y}}_{p}^{M}$	${\bar{y}}_{bk}^{M}$
-0.1	10	2.5734	4.1280	2.5477	0.0712	0.0439	0.1599	0.0987	0.1909	0.1178	0.2060	0.1271	0.2172	0.1340
	20	1.2954	2.0642	1.2824	0.0356	0.0221	0.0799	0.0496	0.0954	0.0593	0.1030	0.0640	0.1086	0.0674
	50	0.5173	0.8223	0.5121	0.0141	0.0088	0.0318	0.0198	0.0380	0.0236	0.0410	0.0255	0.0432	0.0269
	100	0.2590	0.4109	0.2564	0.0070	0.0044	0.0159	0.0099	0.0190	0.0118	0.0205	0.0127	0.0216	0.0134
	200	0.1294	0.2054	0.1281	0.0035	0.0022	0.0079	0.0049	0.0095	0.0059	0.0102	0.0063	0.0108	0.0067
	500	0.0518	0.0821	0.0512	0.0014	0.0008	0.0031	0.0019	0.0037	0.0023	0.0041	0.0025	0.0043	0.0026
-0.3	10	2.5967	3.2711	2.3630	0.0564	0.0407	0.1267	0.0915	0.1512	0.1092	0.1632	0.1179	0.1721	0.1243
	20	1.3082	1.6226	1.1905	0.0280	0.0205	0.0628	0.0461	0.0750	0.0550	0.0809	0.0594	0.0854	0.0626
	50	0.5219	0.6426	0.4749	0.0110	0.0082	0.0249	0.0184	0.0297	0.0219	0.0320	0.0237	0.0338	0.0249
	100	0.2614	0.3207	0.2378	0.0055	0.0041	0.0124	0.0092	0.0148	0.0110	0.0160	0.0118	0.0168	0.0125
	200	0.1307	0.1602	0.1189	0.0027	0.0020	0.0062	0.0046	0.0074	0.0055	0.0079	0.0059	0.0084	0.0062
	500	0.0523	0.0640	0.0475	0.0011	0.0008	0.0024	0.0018	0.0029	0.0022	0.0031	0.0023	0.0033	0.0025
-0.5	10	2.5879	2.4128	1.9409	0.0416	0.0335	0.0934	0.0752	0.1115	0.0897	0.1204	0.0968	0.1269	0.1021
	20	1.3030	1.1789	0.9772	0.0203	0.0168	0.0456	0.0378	0.0545	0.0452	0.0588	0.0487	0.0620	0.0514
	50	0.5198	0.4627	0.3898	0.0079	0.0067	0.0179	0.0151	0.0214	0.0180	0.0230	0.0194	0.0243	0.0205
	100	0.2603	0.2302	0.1952	0.0039	0.0033	0.0089	0.0075	0.0106	0.0090	0.0114	0.0097	0.0121	0.0102
	200	0.1302	0.1148	0.0976	0.0019	0.0016	0.0044	0.0037	0.0053	0.0045	0.0057	0.0048	0.0060	0.0051
	500	0.0520	0.0458	0.0390	0.0008	0.0006	0.0017	0.0015	0.0021	0.0018	0.0022	0.0019	0.0024	0.0020
-0.7	10	2.5683	1.5577	1.3098	0.0268	0.0226	0.0603	0.0507	0.0720	0.0605	0.0777	0.0653	0.0819	0.0689
	20	1.2917	0.7370	0.6587	0.0127	0.0113	0.0285	0.0255	0.0340	0.0304	0.0367	0.0328	0.0387	0.0346
	50	0.5154	0.2836	0.2628	0.0048	0.0045	0.0109	0.0101	0.0131	0.0121	0.0141	0.0131	0.0149	0.0138
	100	0.2580	0.1402	0.1316	0.0024	0.0022	0.0054	0.0051	0.0064	0.0060	0.0070	0.0065	0.0073	0.0069
	200	0.1290	0.0697	0.0658	0.0012	0.0011	0.0027	0.0025	0.0032	0.0030	0.0034	0.0032	0.0036	0.0034
	500	0.0516	0.0277	0.0263	0.0005	0.0004	0.0011	0.0010	0.0013	0.0012	0.0014	0.0013	0.0014	0.0013
-0.9	10	2.5347	0.7060	0.4815	0.0121	0.0083	0.0273	0.0186	0.0326	0.0222	0.0352	0.0240	0.0371	0.0253
	20	1.2725	0.2970	0.2417	0.0051	0.0041	0.0115	0.0093	0.0137	0.0111	0.0148	0.0120	0.0156	0.0127
	50	0.5080	0.1053	0.0965	0.0018	0.0016	0.0040	0.0037	0.0048	0.0044	0.0052	0.0048	0.0055	0.0050
	100	0.2543	0.0505	0.0483	0.0009	0.0008	0.0020	0.0018	0.0023	0.0021	0.0025	0.0024	0.0026	0.0025
	200	0.1271	0.0247	0.0241	0.0005	0.0004	0.0010	0.0009	0.0012	0.0011	0.0013	0.0012	0.0013	0.0012
	500	0.0508	0.0097	0.0096	0.0002	0.0001	0.0004	0.0003	0.0005	0.0004	0.0005	0.0004	0.0005	0.0004

**Table 4:** *RE* of existing and suggested estimators for various values of *ρ*_*xy*_, λ and *n*.

				λ_2_ = 0.05		λ_2_ = 0.25		λ_2_ = 0.5		λ_2_ = 0.75		λ_2_ = 1	
*ρ*_*xy*_	*n*	${\bar{y}}_{p}$	${\bar{y}}_{bk}$	${\bar{y}}_{p}^{M}$	${\bar{y}}_{bk}^{M}$	${\bar{y}}_{p}^{M}$	${\bar{y}}_{bk}^{M}$	${\bar{y}}_{p}^{M}$	${\bar{y}}_{bk}^{M}$	${\bar{y}}_{p}^{M}$	${\bar{y}}_{bk}^{M}$	${\bar{y}}_{p}^{M}$	${\bar{y}}_{bk}^{M}$
-0.1	10	0.6234	1.0100	36.1432	58.6169	16.0938	26.0729	13.4803	21.8455	12.4922	20.2470	11.8480	19.2044
	20	0.6275	1.0101	36.3876	58.6153	16.2127	26.1169	13.5786	21.8448	12.5766	20.2406	11.9281	19.2195
	50	0.6290	1.0101	36.6879	58.7840	13.5774	26.1262	13.6131	21.9194	12.6170	22.9911	11.9745	19.2300
	100	0.6303	1.0101	37.0000	58.8636	16.2893	26.1616	13.6315	21.9491	12.6341	20.3937	11.9907	19.3283
	200	0.6299	1.0101	36.9714	58.8181	16.3797	26.4081	13.6210	21.9322	12.6862	20.5396	11.9814	19.3134
	500	0.6309	1.0117	37.0000	64.7500	16.7096	27.2631	14.0000	22.5217	12.6341	20.7200	12.0465	19.9230
-0.3	10	0.7938	1.0989	46.0407	63.8009	20.4948	28.3792	17.1739	23.7793	15.9111	22.0245	15.0883	20.8905
	20	0.8062	1.0988	46.7214	63.8146	20.8312	28.3774	17.4426	23.7854	16.1705	22.0235	15.3185	20.8977
	50	0.8121	1.0989	47.4454	63.6463	20.9598	28.3641	17.5723	23.8310	16.3093	22.0210	15.4408	20.9598
	100	0.8150	1.0992	47.5272	63.7560	21.0806	28.4130	17.6621	23.7636	16.3375	22.1525	15.5595	20.9120
	200	0.8158	1.0992	48.4074	65.3500	21.0806	28.41130	17.6621	23.7636	16.5443	22.1525	15.5595	21.0806
	500	0.8171	1.1010	47.5454	65.3750	21.7916	29.0555	18.0344	23.7727	16.8709	22.7391	15.8484	20.9200
-0.5	10	1.0725	1.3333	62.2091	77.2507	27.7077	34.4135	23.2098	28.8506	21.4941	26.7345	20.3932	25.3467
	20	1.1052	1.3334	64.1871	77.5595	28.5745	34.4708	23.9082	28.8274	22.1598	26.7556	21.0161	25.3501
	50	1.1234	1.3335	65.7974	77.5820	29.0391	34.4238	24.2897	28.8777	22.6000	26.7938	21.3909	25.3560
	100	1.1307	1.33335	66.7435	78.8787	29.2471	34.7066	24.5566	28.9222	22.8333	26.8350	21.5123	25.5196
	200	1.1341	1.3340	68.5263	81.3750	29.5909	35.1891	24.5660	28.9333	22.8421	27.1250	21.7000	25.5294
	500	1.1353	1.3333	65.0000	86.6666	30.5882	34.6666	24.7690	28.8888	23.6363	27.3682	21.6666	26.0000
-0.7	10	1.6487	1.9608	95.8320	113.6415	42.5920	50.6568	35.6708	42.4512	33.0540	39.3307	31.3589	37.2757
	20	1.7526	1.9609	101.7086	114.3097	45.3228	50.6549	37.911	42.4901	35.1961	39.3810	33.3772	37.3323
	50	1.8173	1.9611	107.3750	114.5333	47.2844	51.0297	39.3435	42.5950	36.5531	39.3435	34.5906	37.3478
	100	1.8402	1.9604	107.5000	117.2727	47.7777	50.5882	40.3125	43.0000	36.8571	39.6923	35.3424	37.3913
	200	1.8507	1.9604	107.5000	117.2727	47.7777	51.6000	40.3125	43.0000	37.411	40.3125	35.8333	37.9411
	500	1.8628	1.9619	103.2000	129.0000	46.9090	51.6000	39.6923	43.0000	36.8571	39.6923	36.8571	39.6923
-0.9	10	3.5902	5.2641	209.4793	305.3855	92.8461	136.2741	77.7515	114.1756	72.0085	105.6125	68.3207	100.1857
	20	4.2845	5.2647	249.5098	310.3658	110.6521	136.8279	92.8832	114.6396	85.9797	106.0416	81.5705	100.1968
	50	4.8243	5.2642	282.2222	317.5000	127.0000	137.2972	105.8333	115.4545	97.6923	105.8333	92.3636	101.6000
	100	5.0356	5.2650	282.5555	317.8750	127.1500	141.2777	110.5652	121.0952	101.72	105.9583	97.8076	101.7200
	200	5.1457	5.2738	254.2000	317.7500	127.1000	141.2222	105.9166	115.5454	97.7692	105.9166	97.7692	105.9166
	500	5.2371	5.2916	254.0000	508.0000	127.0000	169.3333	101.6000	127.0000	101.6000	127.0000	101.6000	127.0000

## 6 Real data illustration

In the present section, we exemplify the suggested estimators using a real data from [36], pp. 31. The real data is based on the density as the study variable *Y* and stiffness as the auxiliary variable *X*. The descriptive statistics are given as follows: *N* = 30, $\bar{Y}=15.47$, $\bar{X}=34666.83$, $\sigma_{y}^{2}=34.023$, $\sigma_{x}^{2}=643900000$ and *ρ*_*xy*_ = 0.89. The execution of the real data illustration is given below.

1. We select 25 samples each of size 5 from the above discussed population using simple random sampling without replacement, which are listed in Table 5 for ready reference.
2. The usual mean estimator $\bar{y}$, usual ratio estimator ${\bar{y}}_{r}$, logarithmic type estimator ${\bar{y}}_{bk}$, memory type ratio estimator ${\bar{y}}_{r}^{M}$ and suggested memory type logarithmic estimator ${\bar{y}}_{bk}^{M}$ are computed using their respective expressions.
3. Using λ_1_ = 0.10 and λ_2_ = 0.25, the *HEWMA* statistic is also tabulated for each sample and the time-varying MSE for different estimators is also listed in Table 5.
4. The *MSE* for the memory type ratio estimator ${\bar{y}}_{r}^{M}$ and the suggested memory type estimator ${\bar{y}}_{bk}^{M}$ is computed as 0.0692 and 0.0109, respectively, with the help of expressions (20) and (27) which shows that the suggested memory type logarithmic estimator dominates the existing estimators.

**Table 5:** Evaluation of the memory type logarithmic estimator consist of *HEWMA* statistic.

Samples of *y*					$\bar{y}$	Samples of *x*					$\bar{x}$	*Q*_*t*_	*Z*_*t*_	${\bar{y}}_{r}$	${\bar{y}}_{bk}$	${\bar{y}}_{r}^{M}$	${\bar{y}}_{bk}^{M}$	$MSE({\bar{y}}_{r}^{M})$	$MSE({\bar{y}}_{bk}^{M})$
19.5	13.6	16.7	21.7	6.4	15.6	32207	18036	49499	47661	8076	31095.8	31823.3	15.5	17.4	16.3	16.9	16.1	0.186	0.109
7	15	15.4	13.6	23.4	14.9	5304	26222	25312	18036	104170	35808.8	6823.1	15.5	14.4	14.6	14.1	14.2	0.359	0.011
21.2	22.8	9.9	13.6	8.3	15.2	48218	70453	14191	18036	7573	31694.2	12537.9	15.5	16.6	15.8	14.9	13.8	0.524	0.014
15.2	8.2	14.5	19.8	8.3	13.2	28028	10728	22148	38138	7573	21323.0	16161.8	15.3	21.5	16.4	15.2	13.6	0.672	0.080
9.9	8.4	7	21.3	23.3	13.9	14191	17502	5304	53045	49512	27910.8	17325.8	14.9	17.4	15.4	14.7	19.5	0.803	0.019
23.4	11	16.7	8.2	15	14.9	104170	19443	49499	10728	25319	41831.8	19721.2	14.7	12.3	13.4	14.4	18.6	0.915	0.011
23.3	14.8	17.4	8.4	9.8	14.7	49512	26751	43243	17502	14007	30203.0	23910.7	14.7	16.9	15.7	14.7	15.1	1.009	0.012
21.2	15.4	9.9	19.5	19.8	17.2	48218	25312	14191	32207	38138	31613.2	25197.2	14.8	18.8	17.8	14.7	15.4	1.089	0.007
11	19.5	25.6	14.5	15.4	17.2	19443	32207	96305	22148	25312	39083.0	26629.9	15.3	15.3	16.3	15.1	17.2	1.157	0.010
23.3	25.6	9.8	14.5	21.3	18.9	49512	96305	14007	22148	53045	47003.4	29278.9	15.7	13.9	16.5	15.5	19.9	1.212	0.014
13.6	21.3	15.4	15	19.8	17.0	18036	53045	25312	26222	38138	32150.6	32526.8	16.3	18.4	17.5	16.3	15.5	1.258	0.021
23.3	6.4	11	15.2	21.2	15.4	104170	8076	19443	28028	48218	41587.0	32640.3	16.4	12.9	14.1	16.3	18.4	1.296	0.016
15	21.7	17.4	9.8	9.5	14.7	26222	47661	43243	14007	14814	29189.4	34181.7	16.2	17.4	15.8	16.2	14.9	1.327	0.107
17.4	14.5	11	24.4	9.9	15.4	43243	22148	19443	72594	14191	34323.8	33285.9	15.9	15.6	15.5	15.9	16.0	1.353	0.054
14.5	9.9	19.5	9.8	9.5	12.6	22148	14191	32207	14007	14814	19473.4	33196.5	15.8	22.5	16.6	15.9	13.6	1.374	0.035
21.2	8.3	25.6	8.4	23.4	17.4	48218	7573	96305	17502	104170	54753.6	31157.5	15.2	11.0	13.6	14.9	15.6	1.391	0.113
22.8	19.5	15	11	9.8	15.6	70453	32207	26222	19443	14007	32466.4	35430.9	15.6	16.7	16.1	15.7	15.6	1.405	0.045
8.2	15.4	16.7	14.5	8.4	12.6	10728	25312	49499	22148	17502	25037.8	34689.5	15.6	17.5	14.8	15.7	14.2	1.416	0.027
8.6	6.4	24.4	16.4	7	12.6	9714	8076	72594	41792	5304	27496.0	32808.3	14.9	15.8	14.1	15.0	14.5	1.426	0.101
8.4	9.5	16.7	6.4	15	11.2	17502	14814	49499	8076	25319	23042.0	31656.8	14.5	16.9	14.0	14.5	13.7	1.433	0.316
9.5	19.5	23.3	8.6	25.6	17.3	14814	32207	49512	9714	96305	40510.4	30283.2	13.9	14.8	16.1	13.9	17.6	1.439	0.112
23.4	15.4	9.9	9.8	13.6	14.4	104170	25312	14191	14007	18036	35143.2	32221.3	14.6	14.2	14.3	14.5	16.3	1.444	0.045
8.6	15.4	8.2	11	21.2	12.9	9714	25312	10728	19443	48218	22683.0	32556.5	14.5	19.7	15.7	14.6	13.9	1.448	0.048
21.2	8.4	17.4	7	19.5	14.7	48218	17502	43243	5304	32207	29294.8	30714.0	14.2	17.4	15.8	14.2	15.0	1.452	0.071
21.3	15	21.2	9.8	25.6	18.6	53045	26222	48218	14007	96305	47559.4	30795.5	14.4	13.5	16.0	14.2	14.1	1.454	0.168

## 7 Conclusion

In this article, we have developed a memory type logarithmic estimator by incorporating the past sample information along with the current sample information in the form of *HEWMA* statistic. The mathematical property, namely, *MSE* of the developed memory type logarithmic estimators is obtained to the first order of approximation. Further, a simulation study is performed over an artificially generated population and presented the simulation results from Tables 1 to 4 that confirm the dominance of the suggested memory type logarithmic estimators over the usual mean estimator, classical ratio and product estimators, the conventional logarithmic estimator envisaged by [3] and memory type ratio and product estimators suggested by [27]. Moreover, a real data illustration is also presented in support of the theoretical results and the results are reported in Table 5 that also show the dominance of the suggested estimators over their entrants. Therefore, the suggested memory type logarithmic estimator is enthusiastically recommended to the survey practitioners for time-based surveys.

In the forthcoming article, we intend to develop the suggested memory type logarithmic estimator using the *HEWMA* statistic under stratified random sampling and ranked set sampling.
